# Exploring the development and maintenance of therapeutic relationships through e-Health support: A narrative analysis of therapist experiences

**DOI:** 10.1177/23992026211018087

**Published:** 2021-06-27

**Authors:** Matthew J Wood, Hannah MN Wilson, Sarah L Parry

**Affiliations:** 1 Manchester Metropolitan University, Manchester, UK; 2 DClinPsy, Kooth PLC, Salford, UK; 3 DClinPsy, Manchester Metropolitan University, Manchester, UK

**Keywords:** Digital, e-therapy, narrative, young people, therapeutic relationship

## Abstract

**Objective:**

Amid COVID-19 disruptions, e-therapy has become even more essential and has rapidly expanded across statutory, private and third sectors to meet growing demands for digital mental health support. A challenge in digital therapeutic care is how to develop and maintain a supportive, collaborative therapeutic relationship, built upon mutual trust and respect; intrinsic values of relationships that are often implied through complex non-verbal cues. Online practitioners are eager to learn how to adapt to online delivery, although platform-specific training is limited. The aim of the current study was to focus upon the therapist experience of online therapeutic relationships with young people, exploring a range of factors through their perspectives, including the impact of anonymity.

**Methods:**

Eight e-therapy practitioners were recruited from Kooth, an online mental health service. Narrative interviews undertaken via Skype facilitated reflective conversational one-to-one discussions, based upon the practitioners’ individual experiences, led by the interviewee. Following transcription and anonymisation, a narrative analysis was undertaken to explore participants’ experiences, perspectives and reflections.

**Results:**

Four analytic layers arose from the narratives, which explored the challenging learning experience of translating existing therapeutic skills to online working, rapidly building therapeutic relationships, managing risk in the online therapeutic relationship, and techniques for maintaining a digital therapeutic relationship.

**Conclusion:**

The study provides novel insights into the flexibility and adjustments therapists can make to improve online interventions and delivery through the development and maintenance of positive therapeutic relationships. Recommendations are also made in relation to platform-specific training, communicative adaptations, risk management and practitioner support.

## Introduction

Online psychotherapy is also called e-therapy, e-counselling or teletherapy. The past decade has seen a rapid increase in online psychotherapy, a flexible approach considered by some to be less stigmatized than face-to-face therapy.^1^ Online psychotherapy is a therapeutic intervention using the Internet to connect service users and mental health professionals.^2^ Cognitive behavioural therapy (CBT) is a NICE-recommended treatment and computerized CBT (cCBT) creates a cost-effective way of improving access to CBT through programmes such as self-help and psychoeducation websites.^3,4^ cCBT can involve the completion of modules of therapy online, guiding the client through stages of learning, reflection and skills practices. Some research combining cCBT and traditional methods reduced contact with the therapist by two-thirds and were equally efficient as face-to-face CBT treatment, while reducing cost by $945 per client.^5^

cCBT programmes translate evidence-based techniques and goals into an accessible multimedia format, which has many benefits. However, questions are raised across the evidence-base as to the role of the therapeutic relationship in this delivery method.^6^ Due to the COVID-19 pandemic, online psychotherapy has become increasingly necessary and popular, making additional research in this area possible.^7^ Generally, therapist’s opinions are reported as positive, especially those from a CBT background. It is suggested that blended or solely online therapy may continue to be used by these therapists in the future.^7^

Research conducted over recent years with young people engaging in cCBT has demonstrated reductions in symptoms of anxiety and depression, showing cCBT to be feasible for young people.^8^ However, as for any psychotherapeutic approach, managing engagement and attrition can be challenging. Within the National Health Service (NHS) implementation of online therapy, up to 50% of adult users of cCBT programmes do not complete the programme,^4,9^ showing similar attrition rates to CBT courses delivered through the Improved Access to Psychological Therapies scheme (IAPT). It is generally recognized that further qualitative enquiry is required to understand these processes.^4,9,10^ Research into online therapeutic work has been largely dominated by cCBT and this body of work provides a useful empirical landscape to building upon. Benefits have been shown for online cCBT but online work also offers opportunities for therapists of other theoretical backgrounds, such as person-centred approaches.^11^

High attrition rates are particularly prevalent in young people, with rates of up to 69% reported.^12^ O’Keeffe et al.^13^ added that adolescents are more likely to prematurely discontinue therapy if the therapeutic approach does not match their desired style. Importantly, a positive therapeutic relationship can be a protective factor against premature attrition within the therapeutic process.^11,14^ Sandoval et al.^15^ concluded that online individual self-help programmes may have higher attrition rates due to the absence of a therapeutic bond. Furthermore, Roos and Werbart^16^ demonstrated that establishing the therapeutic relationship reduced client dissatisfaction and ultimately reduced attrition. These findings were supported by Janeiro et al.^17^ who found that those who begin therapy with a higher therapeutic bond were more likely to successfully complete the course. Finally, Anderson et al.^18^ observed that conflict in the therapeutic relationship would also lead to attrition. Therefore, therapeutic relationships are clearly an important factor to explore, understand and utilize for the effective delivery of online psychotherapies.

One of the main criticisms of online therapy is whether the therapeutic relationship can be built as effectively.^19^ Geller^20^ stated that online therapeutic relationships can be maintained by the creation of a safe and open online environment and Sucala et al.^21^ found that despite previous concerns about building online therapeutic relationships, online therapy was equal to, if not more effective than, face-to-face delivery in creating strong relationships. However, Aafjes-van Doorn et al.^22^ reported therapists explaining difficulty feeling emotionally connected online, yet they reported an overall positive experience of online therapeutic relationships. The growing evidence base is illustrating how online therapeutic relationships can be effective, although therapists continue to voice their concerns^23^ and request further training.^24^ Qualitative research with frontline practitioners could help provide a more nuanced understanding of the therapeutic mechanisms at work, which could foster and nurture positive therapeutic relationships through e-health platforms.

Acknowledging the challenges in building online therapeutic relationships, therapists want to learn and continue to gain experience.^25^ Interestingly, in some studies, therapists have reported that service users found it easier to establish the early relationship online, with trust built without judgement.^26,27^ Optimistically, Trepal et al.^28^ stated that with slight modifications, therapeutic skills could be easily adapted to work online. In addition, person-centred common factors can be used to overcome the loss of physical presence when working online.^11^ For example, adding stylistic choices to text chats such as punctuation and emoticons can help to build a therapeutic relationship. However, losing visual cues such as eye contact can make it difficult for therapists to tell whether their attempts at connection are well received,^29,30^ which may influence practitioner satisfaction and hope regarding outcomes for their client. A person-centred approach to care allows for consideration of the many factors influencing an individual beyond their presenting problem and promotes tailoring work to an individual’s needs.^31^ A person-centred framework could provide space for the modifications required for working successfully online.^28^ Further qualitative research to understand more about the processes at work for practitioners and clients could inform a trans-modal person-centred framework for digital health delivery.

The appeal of newer forms of therapy such as online or text-based chats with younger people is clear, although therapists may need to find new ways to express empathy and warmth through their messages.^32^ Young service users report that the online environment feels safer and less stigmatizing, with the anonymity giving the young person more power in the therapeutic relationship.^33^ For instance, Dunn^34^ proposed that anonymity gave service users more control over when they engage and therefore strengthened agency in the therapeutic relationship. Some research has argued that due to anonymity and the loss of non-verbal communication, the therapeutic relationship cannot develop as comprehensively, but the same study found working alliance scores were higher in the online therapy sample.^30^

Contextually, adolescents prefer to go online for therapy due to the perceived anonymity and as stigma may be decreased,^32,35^ although there remains discrepancy across services as to whether crisis services should be contacted if significant risk is disclosed.^36–38^ The anonymity of online therapy has been associated with greater self-disclosure compared to face-to-face therapy, with service users being more comfortable to discuss a range of topics. However, the impact of this anonymous risk upon the therapist has not been studied to the same extent.^39,40^

Since the outbreak of COVID-19, Geller20 has reviewed the literature to assess the potential impact of the pandemic upon the therapeutic relationship when it moves online, proposing that therapists will adjust and carry forward their new learning post-pandemic. The current study aimed to take this research a step further in real time,^41^ exploring how therapists perceive themselves in employing a range of therapeutic approaches to maintain a person-centred approach in an online therapeutic relationship.^11^ Consequently, a range of counsellors and emotional wellbeing practitioners were asked to reflect and narrate their experiences during the UK lockdown, between April and July 2020, with the objective of informing our emerging knowledge of working therapeutically with young people online. Exploring online practitioners’ narratives of developing and maintaining positive therapeutic relationships, while managing the impact of anonymity through text-based interactions, offers a depth of insight that could guide this rapidly expanding medium of psychotherapy.

## Materials and methods

### Design

The current study employed a qualitative design, suited to researching individual experiences. Ethical approval was gained from the Manchester Metropolitan University Research Ethics Committee^42^ (PsychREC Ref No.: 17155). Prior to data collection, Patient and Public Involvement (PPI) was conducted with a member of staff to discuss the appropriateness of the interview questions. PPI can increase the quality and rigour of the research and has be shown to be beneficial for interview schedules.^43^ The researcher gains new knowledge within the context of the service, via the experiences shared in PPI,^44^ which can then inform the research design and engagement process with participants.

### Participants

Eight online practitioners were recruited as participants via an advert shared through Kooth PLC., a participant sample sufficient to gain rich in-depth data through narrative data collection and analysis,^41,45,46^ with salient themes emerging across the data set.^47,48^ Participants were given a participant number to anonymise their data, enabling them to withdraw from the study if requested. Each participant number was then attributed to a unique code for example., 12 V, which was used to provide further anonymity during the analysis. Participants were eligible if they were over 18, worked for Kooth and had delivered online therapy via Kooth’s Children and Young People’s (CYP) provision. Kooth CYP provides an anonymous online mental health service for people aged 10–25 years old. The nature of Kooth CYP meant that participants would have varied experience of delivering therapy on the platform, as therapy was offered anonymously in optional one-hour slots and could be accessed once or over several sessions (Table 1).

**Table 1.** Online practitioner demographics.

Participant number	Age range (years)	Gender	Job role	Time working for Kooth	Preferred therapeutic approach
101	30–40	Female	EWP	6 months	INT
116	30–40	Female	Counsellor	1 year	Humanistic/INT
120	30–40	Male	Clinical lead/counsellor	6 years	CBT
202	20–30	Female	Assistant psychologist	6 months	CBT and CFT
232	30–40	Female	EWP	1 year	INT
296	30–40	Female	EWP	2½ years	Person-centred
858	40–50	Female	Counsellor	6 years	Person-centred
934	30–40	Male	EWP	3 years	Person-centred

EWP: emotional well-being practitioner; CFT: compassion focussed therapy; INT: integrative approach.

### Interviews

The study used online interviews conducted via Skype to collect data and provided a Qualtrics survey option for participants who wished to participate anonymously.^49^ No participants chose to participate via the Qualtrics survey; therefore, all participants partook through Skype interviews. The epistemological position of social constructionism is suited to considering the socio-cultural context of the participants and their experiences, which aligns with the foundations of narrative approaches.^41,50^ A narrative semi-structured interview method was used as per Fernández-Balboa and González-Calvo.^51,52^ This method uses open questions while keeping the research topic area in mind and asking questions on this area of interest. The balance between structuring the narrative interview to maintain confidentiality and focus on the topic while not interfering with the participants narrative was upheld.^53,54^ Interviews were conducted by the first author, who had received interview training through the university and was supported through the process by the final author.

### Procedure and ethical considerations

Participants were recruited via opportunity sampling following an email distributed through Kooth. Only the first author knew the identity of the participants to reduce potential coercion.^55^ Participants were then sent the Participant Information Sheet (PIS) in advance, containing the details of the study with no deception. Verbal informed consent was sought before the interview commenced as interviews were conducted online and participants were reminded that they did not need to answer any questions they did not want to. The audio recording of consent was recorded via a secure laptop and then stored separately to the research data on the University encrypted storage drive as agreed by the Research Ethics Committee (PsychREC Ref No.: 17155). Interviews were conducted from the home of the first author between June and July 2020. Recordings were then audio recorded and transcribed anonymously, removing any key identifiers before being moved to the encrypted secure University drive. Interviews ranged from 36 min 15 s to 51 min 46 s, with a mean time of 45 min 21 s. The total engagement time for each participant was around 60 min. Transcription was conducted verbatim to include the dynamics between the participant and researcher.^56^

### Analytic approach

Following transcription, a narrative analysis was used to enable the participants’ own sensemaking to be studied.^41^ As stories are the way individuals describe experiences, a narrative analysis allowed for the therapist’s experiences to be explored from within their own social world.^57^ Three levels of narratives were explored in the analysis: the personal, interpersonal and societal.^58^ At the personal level, narratives are constructed to detail personal experiences and the sense of self within those.^59^ The interpersonal level is co-created in the conversation; these are narratives told to entertain or explain to the listener.^58,59^ Acknowledging the impact the researcher has on the data is an important factor in increasing rigour,^60^ especially within this second analytic layer. Therefore, reflective conversations took place between the authors to explore the emerging analysis reflectively. Third, the societal narratives are commonly shared with a community. In this study, the community was one of online therapists.^58^ The societal level acknowledges that narratives are shaped by the context they occur in, mirroring the social constructionist approach.^59^ The aim of any narrative analysis is to explore the individual narrative within the context of its social world, exploring similarities and differences in more depth.^61,62^ As a small, in-depth, narrative study, saturation was not an objective of the research.

## Analysis and results

Emerging from the eight participant narratives were four analytic layers, narrating the learning experience of delivering a therapeutic service online, developing and maintaining online therapeutic relationships. The narratives have been split into parts as per previous research from Jewett et al. and Wilson et al.^63,64^ Part 1 focusses on the learning experience described by online practitioners, Part 2 discusses the way relationships are built online, Part 3 highlights the changes in control and working with risk. Finally, Part 4 concludes the narrative, detailing the experience of maintaining the online therapeutic relationship.

### Part 1 – a challenging learning experience: ‘a steep steep learning curve’ (10A)

Within the first part of the narratives, the experience of working online was consistently described as challenging and a learning experience. With the first descriptions of online work being described as ‘a steep steep learning curve’ (10A) and ‘challenging, but pretty positive’ (21L). These challenges include those that practitioners ‘don’t have in face to face work as much’ (16E). The ‘learning curve’ was shared across the narratives with 17J saying ‘there’s huge amounts of learning in terms of how er how quick you can build relationships with young people’ and 12V also experienced this ‘learning curve’ when working online and stated ‘it can take a while I think to adjust to that’, ‘having to utilize different skills’ (15P), but it can give ‘a very different range of versatility’ (09R). Therapists have reported difficulty in building therapeutic relationships online but show a desire to want to experience learning and not just reject online therapy.^25^

Despite the learning curve, participants could see ‘a lot of the value in it’ (10A) and did not feel that the online experience was more challenging than face to face as ‘I develop my own particular way of working with each person individually’ (12V) and work ‘in a different way’ (15P). This more person-centred view of delivering online therapy has been shown to help therapists overcome the loss of physical presence.^11^ All participants acknowledged challenges within their narratives but 295F felt ‘the challenges are, the clients who are normally challenging for kind of other services, that’s intensified a little bit by the fact they are anonymous’, and the impact of anonymity was a theme running through all parts of the narratives.

### Part 2 – building therapeutic relationships quickly – ‘there’s huge amounts of learning in terms of how er how quick you can build relationships’ (17J)

One of the common narratives shared by many participants was the difference in the speed relationships can be developed online, ‘there’s huge amounts of learning in terms of how er how quick you can build relationships’ (17J), ‘much quicker in my opinion than any face to face’ (15P), continuing the theme of a ‘learning curve’ for therapy online. This has been shown in the literature where Wagner et al.^26^ found high therapeutic alliance scores were found from early points in the online relationship. There were still challenges in building online relationships appearing in the narratives, as ‘things can feel actually quite fast sometimes’ but they were concerned ‘if trust is then still built at the same ratio of speed’ (12V), worrying that ‘it just doesn’t gel’ (21L). When ‘a young person’s quite sceptical erm that can be a barrier’ (15P). Participants outlined narratives explaining how they bypassed some of these challenges from ‘connecting beyond a human level’ (21L) and making adjustments to practice, making it ‘easier for me to connect therapeutically online because of those changes’ (09R). This aligns with the person-centred approach to delivering online therapy, making adjustments for each individual^11^ that help to bypass the challenges provided by online therapy, such as relationship building feeling ‘quite fast’ (12V).

The control of how the therapeutic relationship was built is with the service user; ‘it’s down to the individual practitioners about how much we build and how much we make the young people feel, relaxed’ (295F), with the service users giving ‘the permission for us to build that relationship’ (15P). Geller^20^ found that creating the relaxed and safe environment talked about by 295F, impacted how relationships could be developed and maintained. Three participants described the experience of building fast relationships with more concern, feeling ‘a bit taken aback by it sometimes’ (12V). The relationships can be ‘quicker established’ (12V). 16E described, ‘I think my experience has been that it’s harder to form a therapeutic relationship’ and tried to detail why they felt relationships were quicker built online saying ‘they share erm kind of more quickly in online’, it keeps ‘coming and coming’ (15P). This is supported in the research where anonymity of online therapy has been shown to increase speed and frequency of disclosure.^39^

Participants were aware of this on the societal level of their narrative, saying they knew that ‘other people have kinda said oh actually you know, you can build the relationship in the same way, but for me that’s not what I’ve, found’, it can take a ‘longer time to build a therapeutic relationship online’ (10A) and despite growing evidence for the effectiveness of online therapeutic relationships, some therapists do still show concerns from their own experiences.^23^ It is important to note that factors such as the speed and frequency of disclosure,^39^ and the need for modification to therapeutic relationship building techniques, can be a difficult adjustment for some practitioners.^28,65^

### Part 3 – risk and control in the online therapeutic relationship: ‘they’re more in control’ (10A)

One of these main concerns that was common in all narratives was the challenge of working with risk online, alongside the change in power within the therapeutic relationship. Sometimes service users come online and ‘want an active kind of response’ (10A) but often for the online practitioners, what the ‘limitations are in terms of working with risk’ (16E) lead to ‘frustration’ and feeling ‘angry’ (21L) as therapists ‘wanna protect’ (295F). This is an ethical dilemma raised in the research as anonymous clients make providing emergency services and gaining contact information difficult.^66^ Risk can change the ‘dynamic’ and being too ‘heavy handed’ can ‘derail er that therapeutic relationship’ (09R). An example of this was shown in the narrative of 16E, ‘we had a safeguarding issue er and in the end she didn’t come back after that’. Patients with high risk often struggle to build strong therapeutic relationships but the perception of the relationship has been shown to impact on risk of suicidal ideation for example.^67^ Many descriptions of working online with risk were shared across all the narratives, from ‘extremely challenging’ and ‘exhausting’ (12V) to ‘helpless’ (21L) but 15P believed the anonymity allowed for ‘a more robust piece of work’.

The impact of risk and anonymity was discussed across the narratives, and one common factor was a ‘power balance’ (12V), and when referring to the service user, there is a ‘feeling that ‘they’re more in control’ (10A). Participants detailed how service users had more control over ‘sharing their details’ (12V) and ‘whether to provide information’ (21L), with therapists ‘looking to encourage them to er give us their details’ (09R). Some online practitioners experienced this lack of control more negatively however, describing how it can feel ‘stilted’ (16E) and leave them with ‘a huge bag of helplessness’ (21L), with 15P feeling that ‘in a face to face setting it’s easier for us to just kind of, manage the session a little bit’. This was an experience shown in the past research, with therapists feeling online clients had more power and control over the time, location and attendance and had an increased control over disclosure, which was beneficial for the therapeutic relationship.^34,68^ Through the societal level of the narratives, support for this was explained such as ‘the first point of call you would erm let the senior practitioner know’ (12V) and ‘having the rest of the team there, on a separate platform’ which was ‘incredibly valuable’ (09R). The research supports this use of online supervision for maintaining professional performance and development, but questions if it is limiting for the supervisor in terms of practicality and technical issues.^69^

### Part 4 – maintaining an online therapeutic relationship – ‘it makes them feel a little less kind of erm you know just standalone sessions each time’ (17J)

Two participants described experiences of finding difficulty maintaining online relationships, from the less defined boundaries of online therapy ‘I guess they expect you to be around a lot more and so that can make it difficult, with the the therapeutic relationship’ (10A) to feeling ‘removed’ and ‘harder even if they have had multiple sessions’ (16E). It is important to ‘keep that engagement going’, sending a ‘follow up message’ (15P) as ‘once actually you’ve found out some information about them, found out kind of their story slightly more, then actually I think anonymity to me doesn’t seem to kind of affect the relationship really’. This is in line with research from Roos and Werbart^16^ who found that if the alliance can be established early in the relationship, it can predict maintenance. 12V and 09R had not experienced any major differences in maintaining online relationships, giving an example of working for ‘at least (2.0) four five months now and and still continuing’, saying ‘I don’t feel sort of online kind of erm effects that in in any way’, with ‘no change there’, feeling online relationship maintenance was ‘relatively the same’ (09R). Geller^20^ outlined how creating the environment of continuation and safety to share can lead to the maintenance of online relationships.

The narratives detailed techniques to maintain the online therapeutic relationship, ‘you might put something in the case notes like I’ll ask this next time, and so in the in the chat then you can use that as a prompt’ (17J), ‘because, the transcripts are stored we can kind of review them after session before we we talk again next week’ (15P). This continuity has been shown to be effective in aiding in maintenance of the therapeutic relationship.^70^ The use of emojis was described as a ‘way to connect’ (09R) in an online therapeutic relationship, as they can ‘relax the atmosphere’ (17J) which has been shown to impact online relationships positively.^20^ Therapists can still ‘watch your tone in text’ through ‘capitals or emojis’ (17J) which was hypothesized by Manfrida et al.^29^ However, some participants did raise concern that these techniques are ‘too simplistic’ (10A) and emoji use ‘blurs the boundaries’ (16E) of the therapeutic relationship. The narratives describe a need for modifying traditional therapeutic skills for online work but raised questions on their effectiveness.^65^

## Discussion

The current study aimed to enhance understanding of therapist’s experiences of developing and maintaining online therapeutic relationships through text-based interactions. The narrative analysis of eight participant interviews revealed four analytic layers. *A challenging learning experience* covered the learning curve and adjustments therapists made when working online. *Building therapeutic relationships* discusses the speed of online therapeutic relationships and the speed of disclosures. *Risk and control* in the online therapeutic relationship outlined therapists experience of having less control of the therapeutic relationship and the difficulty that can bring when working with risk. Finally, *maintaining an online therapeutic relationship*, details techniques therapists used to maintain online therapeutic relationships and whether therapists experienced differences in the use of therapeutic techniques from traditional face-to-face work. The experience of online practitioners developing and maintaining online therapeutic relationships has been discussed in detail through the narratives, highlighting facilitators and barriers to the process and experience, complimenting previous literature that shows an overall positive experience, accompanied by some concerns around safety and responsibility.^7,23^

The current study also expands knowledge in that attrition rates were not discussed by the participants, although this is a frequent topic of concern in the literature,^4,9^ especially with young people.^12^ However, this was not a frequent issue experienced by the online practitioners within their narratives; only mentioned once within Part 3 within the larger context of risk. Managing risk was a significant challenge outlined by therapists within their narratives.^18^ Research has begun to suggest that risk is not as much of a concern as it was at the beginning of online therapy, although clearly still a challenge for the individual therapist experience.^71^The idea that online therapy gives more control to the young person was consistently supported by the narratives, ‘they’re more in control’ (10A). Research has not previously discussed in detail the impact this has upon the therapist experience, with some therapists reporting difficulties, feelings of helplessness and being stuck (21L, 16E). Due to anonymity, online practitioners reported occasions where they were not able to engage other services in the handling of risk, where they typically would have; the biggest challenge in their experiences raised by the current research.

Further, equivocal findings in the literature exist around whether a strong therapeutic relationship could be developed online,^19^ although the results of the current study act in support of Sucala et al.^21^ and Geller^20^ as online practitioners described relationships as being similar but developed more quickly (09R,12V) as per Fletcher-Tomenius and Vossler.^27^ This quicker establishment may be due to rapid self-disclosure,^39^ which was a theme in the narratives, with therapists saying they could be taken aback by how quickly self-disclosure can occur online (15P,12V). Quicker self-disclosure is not necessarily a sign of a good therapeutic relationship, it may be that as literature has suggested, quicker self-disclosure online is due to anonymity^72^ or as Raufman and Shahak^73^ found increased self-disclosure online without visual anonymity, it may suggest that it is the online nature of the work, rather than anonymity, which may be the crucial factor in quicker self-disclosure.

Creating a safe online environment can also maintain the therapeutic relationship,^20^ adjusting to the online dynamic to best help service users feel relaxed (295F, 09R). This supports Trepal et al.^28^ as slight modifications were made to maintain an online therapeutic relationship (15P). Both 09R and 295F described person-centred elements within their therapeutic work, and it may be that this person-centred framework^31^ provided the opportunity for modifications and flexibility to the individual need online. Overall, the findings of this study suggest that the principles of a person-centred approach are more integral to the development and maintenance of the therapeutic relationship than features of a specific modality, such as CBT. It would however be interesting to conduct a mixed methods study with practitioners and young clients to explore the roles of the therapeutic relationship and features of therapeutic modalities in unison, exploring the complementary or incongruous aspects of what may be an integrative or trans-modal approach, and the impact upon client outcomes.

The narratives conclude that the maintenance of a therapeutic relationship is not particularly impacted by online anonymity. There was no change reported by some therapists, but two participants did report maintenance being harder despite many sessions together (16E, 09R). This may explain why the past literature has shown concerns from therapists^23^ but the current study shows online therapy to be capable of generating the same results as face-to-face support, as per Sucala et al.^21^

Previous research has queried whether online therapists would be required to make modifications to their standard techniques to work effectively online.^28^ It has been proposed that increasing person-centred techniques could help to build online therapeutic relationships^11^ and stylistic additions to text chat such as emoticons, punctuation and capitalisation could be a way of overcoming the loss of physical non-verbal cues.^29^ The question of whether these techniques would be received in the same way by service users and whether therapists would find these techniques as effective was considered by Simpson,^30^ although limited research with therapists has impaired progress in this area. The current study demonstrates that techniques outlined by Manfrida et al.,^29^ such as connection through emojis and grammatical techniques were beneficial (17J, 09R). Conversely, some therapists worried that these techniques may blur the boundaries of the relationship and simplify the dynamic (10A,16E), providing new insights into the experience of online therapists working to develop and maintain online therapeutic relationships.

Békés and Aafjes-van Doorn^7^ highlight that qualitative research into the experience of therapists working online would be beneficial and the current study contributes novel insights to address the scarcity of research in this area. However, the sample for the current study was from one service, so is unlikely to be representative of therapist experiences across the sector. That said, the novel findings provide new avenues for exploration and highlight the heterogeneity in therapists approaches and experiences. The nature of Kooth meant that their practitioners are located around the country and therefore online interviews allowed for the best access to this sample.^74^ However, the nature of online interviews raised the possibility of ‘lagging’ or technical issues that disrupt the flow of the interview.^75^ A larger scale study could provide opportunity for a range of data collection methods to be used.^76^ The sample for the current study was 75% female, which may mean female therapist experiences were over-represented. However, findings from the UKCP (UK Council for Psychotherapy) show that 74% of their members are female, suggesting the sample for this study is representative of therapists nationwide.^77^ The desire for online therapy continues to expand and there is need for platform specific training to aid with this^24^ as current online services often maintain traditional techniques and require modifying to suit the platform.^65^ Such training could be delivered by therapists with direct experience to share their implicit learning, supported by service users to represent both perspectives of the process.

## Conclusion

This study is one of the first to demonstrate how therapists delivering e-therapy perceive that young people can feel a greater sense of autonomy in online therapy, compared to face-to-face support, although this anonymity can leave online practitioners feeling vulnerable as to how they manage aspects of risk. Participants described how therapeutic relationships can be built as effectively online as face-to-face, often developed faster, partly due to rapid disclosures from service users. Participants detailed the use of modified techniques to overcome the loss of a physical presence, although there was significant variety in the use of techniques across the sample. In conclusion, practitioners with experience of online working have a great deal of learnt expertise they could share, although also express a desire for platform-specific training to help them modify their techniques, aligning to online interventions. Consequently, one method of training may be to facilitate reflections on positive actions, reassuring therapists of their own good practice and sharing those practices across the training group. Future research could adapt the methodology of this study to provide further insights based upon these novel nuanced findings, such as through a mixed-methods content analysis through a larger multi-service sample. Upholding principles of a person-centred framework seems helpful for online working, supporting a range of therapeutic techniques. The focus on the therapist experience can inform training and delivery for online therapy, guiding future provision and supporting therapists through this rapidly developing landscape.
